# Incidence of systemic autoimmune myopathies and their risk of cancer in Leeds, UK: an 11-year epidemiological study

**DOI:** 10.1093/rap/rkac023

**Published:** 2022-03-28

**Authors:** Benoit Jauniaux, Memie Alexander, Azzam Ismail

**Affiliations:** 1 School of Medicine, University of Leeds; 2 Department of Histopathology, St James’s University Hospital, Leeds, UK

**Keywords:** systemic autoimmune myopathies, myositis, PM, DM, malignancy, epidemiology, incidence rate, relative risk

## Abstract

**Objectives:**

The aims were to identify all incident adult cases of systemic autoimmune myopathies (SAMs) in the city of Leeds, UK, and to estimate the risk of cancer in SAMs as compared with the general population.

**Methods:**

Cases of SAMs were ascertained by review of all muscle biopsy reports from the Neuropathology Laboratory. A review of medical records was undertaken for each case to review the clinical diagnosis and collect epidemiological data such as age, ethnicity, sex and comorbidities, including cancer. Leeds denominator population numbers were publicly obtainable.

**Results:**

A total of 206 biopsy reports were identified and, after review, 50 incident cases were included in the study between June 2010 and January 2021. Of the 50 cases, 27 were male and 23 were female. The mean incidence rate of SAMs in Leeds throughout the study period was 7.42/1 000 000 person-years. The proportion of SAMs cases with a confirmed malignancy was 22%. Compared with the general population, the relative risk of cancer was significantly greater in the SAMs population (31.56; *P* < 0.01).

**Conclusions:**

The incidence rate of SAMs in Leeds was consistent with data from previous literature; however, disagreement exists between different methods of SAMs case inclusion due to varying clinical criteria and definitions. SAMs are associated with an increased risk of cancer, but the pathogenesis of this relationship still requires investigating. This study supports the practice of malignancy screening and long-term surveillance in patients with SAMs.

Key messages

- This study provides incidence data to complement existing reviews on systemic autoimmune myopathies (SAMs).
- The study also supports the practice of cancer screening and long-term surveillance in SAMs.

## Introduction

Systemic autoimmune myopathies (SAMs) represent a heterogeneous group of immune-mediated syndromes characterized by inflammation within the skeletal muscles [1]. SAMs have most commonly been classified as DM, PM and IBM [2]. More recently, SAMs have included further subtypes such as immune-mediated necrotizing myositis (IMNM), antisynthetase syndrome, unspecific myositis and overlap myositis [3]. SAMs may be classified according to biopsy results, presence of antibodies, blood tests or clinical features. Clinicians tend to use the Bohan and Peter criteria [4] or the recently proposed EULAR and ACR criteria to verify SAMs [5]. Briefly, the criteria apply scored variables and the gross sum of the scores is converted into a probability of a SAM diagnosis. These diseases can be severely impairing and muscle weakness, dysphagia or extramuscular manifestations such as interstitial lung disease or arthritis may cause significant impairment in these patients.

SAMs are rare diseases and the incidence varies greatly across studies, from 1.2 to 66 cases of SAMs per 1 million persons per year [6], usually twice as common in females as in males [7]*.* While a number of UK centres have contributed to collaborated international research, there has only been one published report, based solely in Salford, investigating the incidence of SAMs in the UK [8]. The epidemiology of SAMs in the UK remains relatively unknown. The study of myositis can reveal important clues to its aetiology, disease associations and identification of risk factors and comorbidities. One of the major contributors to morbidity investigated in SAMs is malignancy-associated [9], with the subtype DM showing the strongest association with cancer [10, 11]. The pathogenesis between this relationship is still unexplained, however, some theories suggest SAMs may have a role as paraneoplastic syndromes [9–16]. A diversity of cancers is observed in SAMs, with significant variation depending on geographic region [17]. Leading sites are the ovary, lung, gastrointestinal tract and breast [11, 18, 19], however, further research is needed to verify this across different populations. Furthermore, the exact effect size of the potential correlation between SAMs and cancer remains inconclusive [2]. In particular, most studies have had too few SAMs cases with each cancer type to focus on the association with specific cancers and provide definitive conclusions [11].

Interrogation of differing geographically diverse populations can assist in constructing a more complete picture of underlying disease patterns in SAMs. To date, due to scarce published reports, there is still very little detail on the incidence of varying clinical subtypes of adult SAMs in the UK, and there have been no studies focussing on the population of Leeds served by St James’s Hospital (SJH), Leeds Teaching Hospitals Trust. SJH is a major centre for SAMs in the UK, located in Leeds, which has a population of 630 000 inhabitants >18 years of age. The rarity and heterogeneity of SAMs are contributing factors to insufficiently powered studies on the epidemiology of the diseases. Understanding the epidemiology of these rare SAMs in the UK could help experts develop accurate classification systems and identify SAM subtypes and their associated comorbidities such as malignancy.

There were two primary aims to this study. The first was to establish the epidemiology of SAMs in Leeds from 2010 to 2021. Further information on the epidemiology of SAMs patients will hopefully provide clinicians with up-to-date data that could be used when making decisions on new guidelines, diagnosis and management of SAM subtypes. The study’s second aim was to estimate the risk of cancer in SAMs as compared with the general population. This could provide insights into the significance of any associations of SAMs with cancer, providing a basis for further research into understanding its pathogenesis.

## Methods

This retrospective observational cohort study was used in the assessment of patients presenting to SJH with systemic autoimmune myopathies between June 2010 and January 2021. Approval for the conduct of the project was granted without a recommendation to seek more formal National Health Service (NHS) Health Research Authority ethics authorization, in keeping with local policy. The study used previously obtained data that were routinely collected as part of patient care. No changes were made to patient care and no patients were approached or required for consent. All data were stored anonymously and securely. There were no conflicts of interest.

### Denominator population

Leeds is a city within West Yorkshire, UK, comprising a spectrum from densely populated areas to open rural space. The annual total mean population is publicly available (Supplementary Table S1, available at *Rheumatology Advances in Practice* online), stratified annually by age and sex, along with annual total cases and incidence rates. Persons <18 years old were removed from all calculations. Annual cases of cancer in Leeds are also made publicly available.

### Cases

The study included patient cases presenting to SJH between June 2010 and January 2021 with a clinical diagnosis of SAMs. Patients were included if they had at least one muscle biopsy that had been assessed by the laboratories of SJH and a definite diagnosis of systemic autoimmune myopathy had been confirmed in the patient notes as a result of all investigations. Most patients received an MRI, electron microscopy analysis and blood tests for antibodies. All patients <18 years of age at the time of SAM diagnosis were excluded. There were no further exclusion criteria, as the objectives of the study were to represent the patient population served by SJH.

**Fig. 1:**
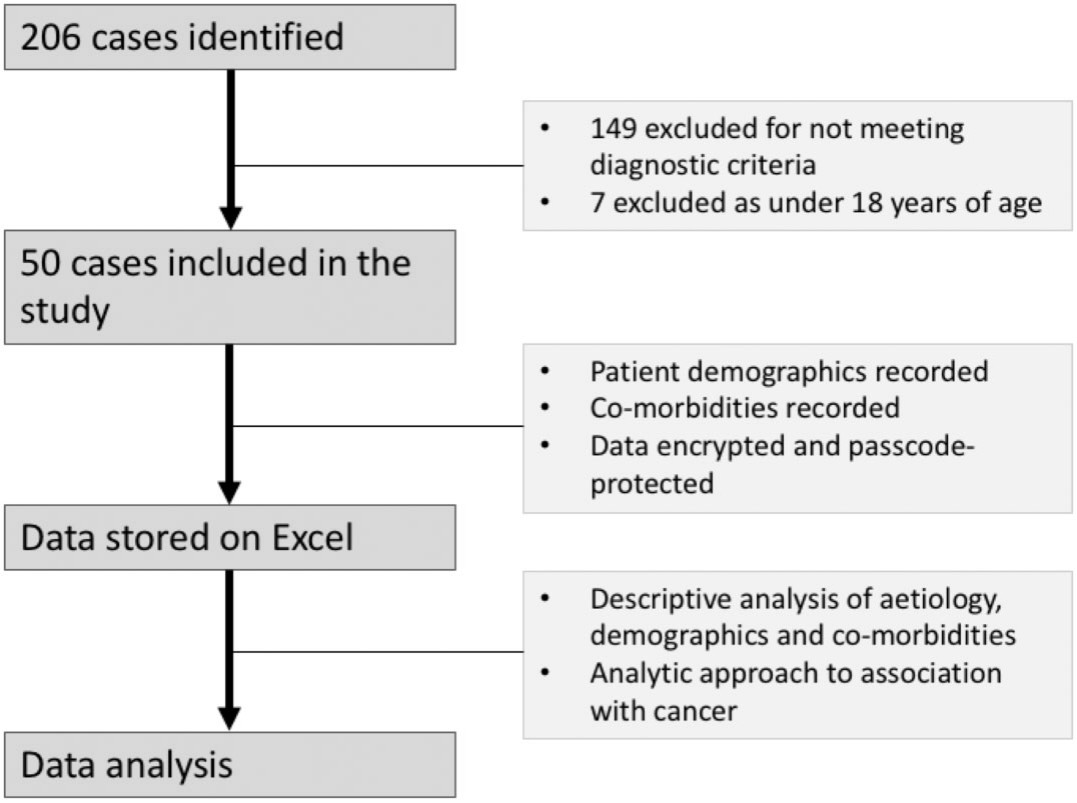
Summary of the methods

In total, 206 cases were selected for potential eligibility from a systematic search of muscle biopsies recorded on the local medical records database (Fig. 1). A manual review of all patients was undertaken using PPM+ NHS software, the SJH patient database, to confirm the results of investigations, diagnosis of SAMs and specific subtypes if available. Diagnoses of myositis were confirmed by comparing biopsy findings with clinical diagnoses and autoantibodies to ensure consistency in the findings. Exclusion criteria were then applied. This resulted in 50 cases being included in this study. Access to all data was encrypted on a passcode-protected Excel 2018 (version 16.16; Microsoft, Redmond, WA, USA) document.

Cancers were manually searched for in individual patients using PPM+ and general practitioner records. Any findings of confirmed malignancies within 10 years before or after the date of the SAMs diagnosis were reported and specified. Any comorbidities, past or present, were also recorded. The patients’ age, ethnicity and sex were recorded. All data were recorded into Excel. For each case, the date of confirmed diagnosis, patient age, ethnicity and sex were recorded.

### Statistics

The adult population for each mid-year period was used as the denominator for that respective year. Incidence rates were presented as numbers per million persons per year in relation to the denominator population and stratified by age and sex. Mean incidence rates were given, accompanied by 95% CIs in parentheses. Means and s.d.s were calculated for descriptive, continuous data that followed normal distribution. Sex-adjusted relative risks (RRs) of cancer were calculated for those with SAMs *vs* the denominator population. Statistical differences between independent categorical variables were calculated using the chi-squared test and presented as exact *P*-values. Statistical analyses were performed using SPSS version 26 (IBM, Armonk, NY, USA) and Excel 2018.

## Results

### Clinical characteristics

There were 50 confirmed incident cases of SAMs between June 2010 and January 2021. Relevant individual patient information is summarized in the Supplementary Table S2, available at *Rheumatology Advances in Practice* online. Table 1 presents the frequency and proportion of different subtypes of SAMs identified in this study. This included 12 PM cases, 10 DM cases, 6 IBM cases, 4 statin-induced autoimmune necrotizing myositis cases and 2 IMNM cases. Sixteen cases were confirmed as SAMs but insufficient information was provided to classify them into subtypes.

**Table 1:** Frequencies and proportion of subtypes of SAMs

Subtype	PM	DM	IBM	Other^a^
Frequency	12	10	6	22
Percentage	24	20	12	44

a Necrotizing (*n* = 2), statin-induced (*n* = 4), not specified (*n* = 16).

**Fig. 2:**
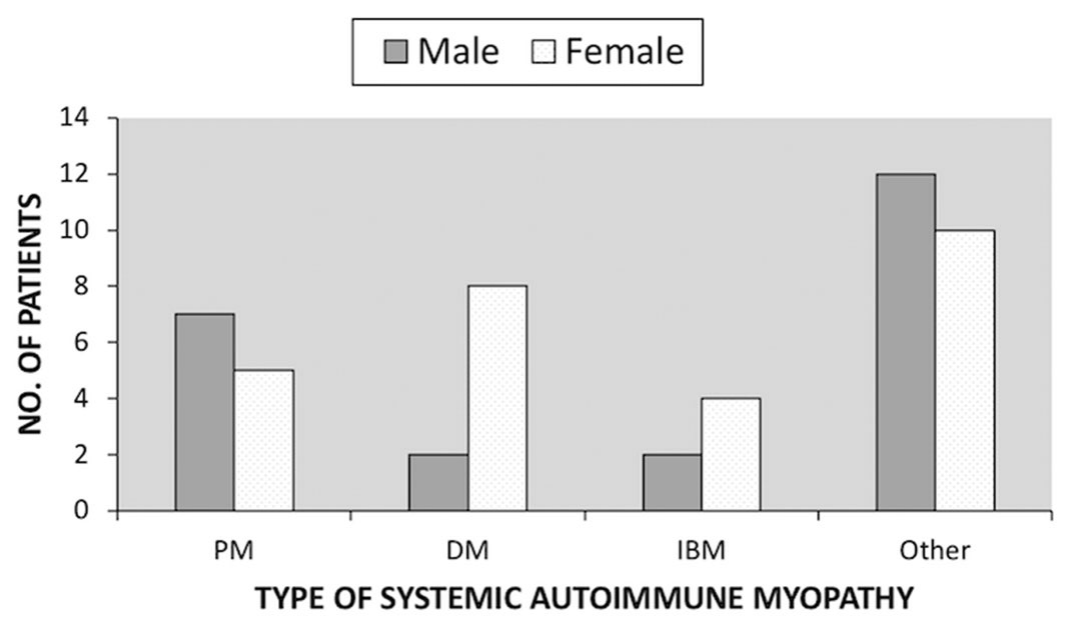
Systemic autoimmune myopathy subtypes by sex

Of the 50 incident cases, 27 were male and 23 were female. Comparing subtypes (Fig. 2), 58% of PM cases were male, 80% of DM cases were female, 67% of IBM cases were female and 55% of the ‘other’ cases were male. A total of 82% and 8% of all cases were of white and Pakistani ethnic origin, respectively.

**Fig. 3:**
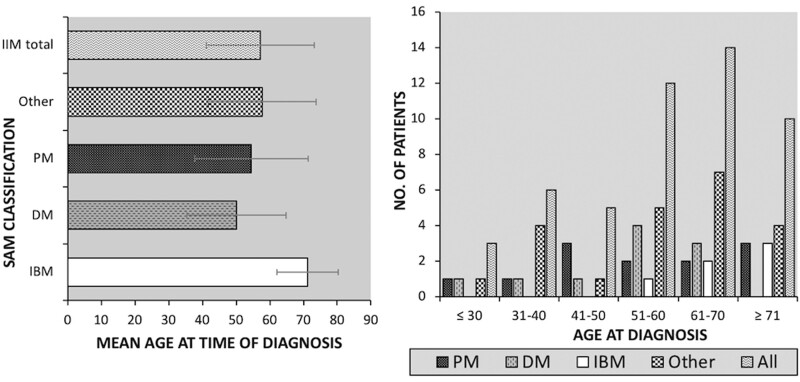
(Left) Mean age at time of SAM diagnosis. (Right) Distribution of SAM subtypes by age

The mean age at disease onset was 57.2 years (s.d. 16) and 52% of cases were 51–70 years of age at the time of disease onset (see Fig. 3). There were no cases of IBM found in persons <59 years of age and the oldest mean age was 71.2 years (s.d. 9). PM was most common between 41–50 years (*n* = 3) and >70 years (*n* = 3). No cases of DM were first diagnosed at >70 years of age.

Individual and summarized data on comorbidities are provided in Supplementary Tables S2 and S3, available at *Rheumatology Advances in Practice* online. Twelve of 50 had hypertension, 5 had interstitial lung disease, 4 had OA or RA and 4 had gout. Four of 12 PM cases had interstitial lung disease. Two of 10 DM cases also had scleroderma. Fourteen individual malignancies were identified in 11/50 cases, 8 of which were found in the 10 DM patients. This included four colon cancers (3/10 DM cases), two basal cell carcinomas of the skin, two pancreas cancers, one squamous cell carcinoma of the skin (in a DM case) and one each of liver, breast, endometrium, lymph node and prostate cancer.

A total of 18 patients (36%) had one or more recorded antibodies on the patient database (Supplementary Table S2, available at *Rheumatology Advances in Practice* online). Anti-Ro52 and ANCA were the most common autoantibodies (nine and six, respectively, among all 50 patients).

### Incidence of SAMs in Yorkshire

**Fig. 4:**
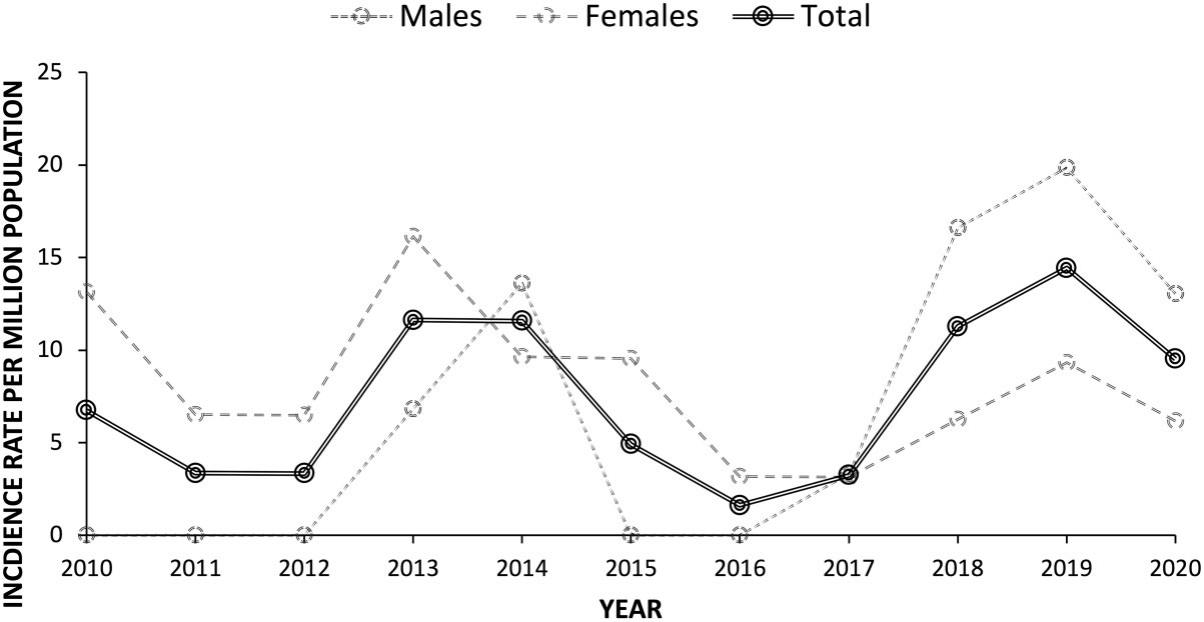
Sex-adjusted annual incidence rates for all SAM patients in Leeds

**Fig. 5:**
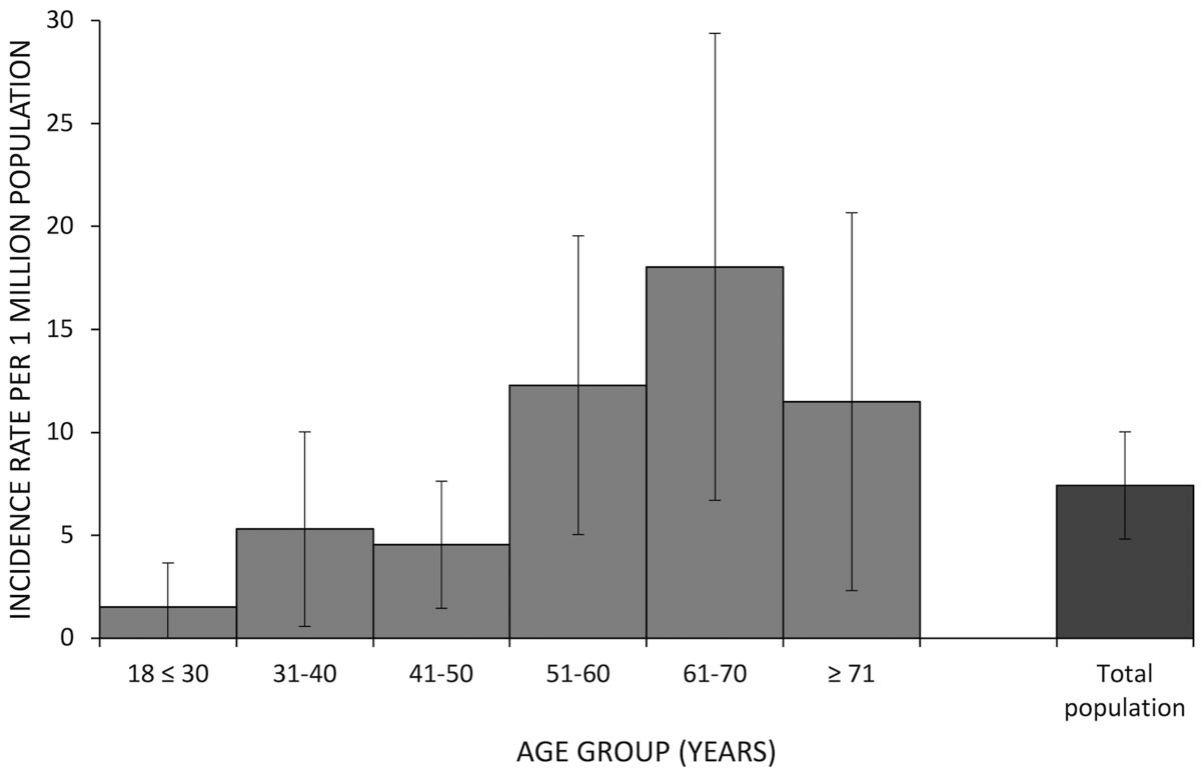
Averaged age-adjusted incidence rates for all SAM patients in Leeds s.e. bars represent the upper and lower CIs at each age group.

The mean incidence rate of SAMs in Leeds throughout the 11 year study period was 7.42 (CI 4.81, 10.03)/1 000 000 person-years (py). The incidence rate for females was higher than for males [8.13 (CI 5.79, 10.47)/1 000 000 py *vs* 6.66 (2.10–11.22)/1 000 000 py, respectively]. Stratifying by calendar year, the highest annual incidence rate was 14.4/1 000 000 py in 2019, but the incidence rates varied greatly each year and by sex (Fig. 4). Annual incidence rates by subtype of SAMs were included in Supplementary Table S4, available at *Rheumatology Advances in Practice* online. Age-standardized incidence rates varied across age groups, generally increasing to and peaking at the 61–70 year age group [18.04 (CI 6.7–29.38)/1 000 000 py] (Fig. 5). The incidence rate was lowest in those <30 years of age [1.53 (CI 0, 3.66)/1 000 000 py]. There was an incidence rate of 11.49 (CI 2.32, 20.66)/1 000 000 py for those >70 years of age.

### SAMs and cancer

The proportion of cancer was 60% in the patients with DM and 8.3% in those with PM (Supplementary Tables S2 and S3, available at *Rheumatology Advances in Practice* online). The proportion of SAMs cases with a confirmed diagnosis of malignancy was 22% (malignancy reported in 11/50 patients). Comparing sex, the frequency was higher in females than males (37% *vs* 21%, respectively). In the denominator population, the mean frequency of cancer was 0.69% (4300/621 018 persons). Comparing males and females, the mean frequency of cancer was 0.75% and 0.65%, respectively. Compared with the total population of Leeds, the RR of cancer in SAMs was 31.56 (CI 18.6, 53.5); in males it was 23.12 (CI 9.5, 56.4) and in females it was 40.15 (CI 21.2, 76.1). There was a significantly greater proportion of cancers found in the SAMs patients compared with the denominator population (*P* < 0.01). The RR of cancer within the cohort, comparing SAM subtypes DM to PM, was 7.2 (CI 1.03, 50.28). Compared with the total population of Leeds, the RR of cancer in DM alone was 86 (CI 51.62, 143.56). The average number of years between a SAM diagnosis and a following diagnosis of cancer was 3 years.

## Discussion

The mean incidence rate for SAMs in this study was 7.42 (CI 4.81, 10.03)/1 000 000/py, representing a similar rate to estimates from other geographical regions [19–21]. A recent systematic review by Meyer *et al.* [20] looked at 46 articles published between 1966 and 2013 and found a mean incidence of 7.98/1 000 000/py ranging from 1.16 to 19/1 000 000/py. The wide range in incidence rates may be explained by the authors reporting limitations to their systematic review due to methodological heterogeneity, in particular the varying methods of SAMs classification used. Only 4/46 studies reported SAMs subtype frequencies and proportions, some of which were based on Bohan and Peter criteria, some on the International Classification of Diseases and others either not reported or study specific. Studies using Bohan and Peter criteria, or requiring cases to be biopsy verified, all reported strikingly similar results to those in the present study. For example, a study from Sweden and one from Australia both required biopsy-verified cases and presented estimates of 7.4 and 7.6/1 000 000/py, respectively [21, 22]. More recently, a large national study in Sweden found a higher incidence rate of 11 (IQR 10–12)/1 000 000/py, however, they only used coding searches to include cases and omitted a manual review of results [6]. Without a manual review of cases, studies are more likely to include false positives. Døbloug *et al.* [23], in a 2015 Norwegian study, estimated an annual incidence of 6–10/1 000 000/py in a Norwegian population, although they did not report the incidence restricted to adults and IBM subtypes were excluded from the analysis. Parker *et al.* [8] studied the incidence rates of SAMs in Salford, UK, between 2007 and 2016. Their study estimated a very high incidence rate of 17.60/1 000 000/py, however, it was a particularly small epidemiological study—32 cases identified in a denominator population of 190 000 persons. Furthermore, it was the only study using the newly proposed EULAR classification system. The study contained only 13/32 biopsy-verified cases due to local clinical practice procedures. The incidence rate in the present study was highest in females [8.13 (CI 5.79, 10.47)/1 000 000/py] than in males [6.66 (CI 2.10, 11.22)/1 000 000/py]. The average age at the time of diagnosis was 58.5 years. These findings were consistent with Meyer *et al.* [20] and recent studies [5, 7]. Incidence rates in the present study increased with age until a peak at 61–70 years. Meyer *et al.* [20] and Parker *et al.* [8] did not present age-adjusted incidence rates, however, the peak age of incidence ranged from 50 to 79 years in other studies [6, 24]. Interestingly, the present study found that the oldest group of patients were the IBM subtype, with a mean age of 71.2 years (s.d. 9), which is consistent with findings in other studies [21, 25].

The heterogeneity of methods and classifications used in other studies is likely responsible for the considerable variance in results [5, 26]. It is highly possible that studies using coding searches alone are overrepresentative of the true incidence rate. Studies requiring biopsy-verified cases, such as the present study, may increase the specificity of results, but they can be underrepresentative since not all SAMs patients are subject to muscle biopsy or present typical biopsy findings. Advances in the comprehension of the pathogenesis of SAMs have contributed to better recognition of SAMs subtypes and new sets of diagnostic criteria have been developed that take into account advances in histopathology and serum autoantibody detection [27], although there still remains a strong need to revise and reclassify [20]. For example, the present study looked at all comorbidities of every patient. SAM is a multisystemic disease [25] and can affect various organs (10% of SAMs cases in this study also suffered from ILD).

The antibodies found in the present study are supported by previous literature [27, 28, 32], in particular the high proportion of anti-Ro52 and ANCA, however, the frequencies may be underrepresented due to missing data and the retrospective limitations of the study design. Furthermore, due to the histopathological focus of SAM diagnoses, the antibody panel lacked several antibodies, cN1A and HMGCR, two important antibodies associated with IBM and IMNM, respectively. It also lacked DM-specific antibodies such as anti-Mi2, anti-MDA5 anti-NXp2. These antibodies are used for characterization of patients and serve a prognostic purpose in defining associations with different complications and pathologies. Nevertheless, anti-Ro52, found in 18% of the SAMs patients in this study, was also the most frequent antibody in other studies [28]. This is of particular interest, as Chung *et al.* [28] also found that anti-Ro52-positive patients had a higher frequency of ILD than negative patients. Unfortunately, the frequencies were too small for analysis in the present study.

The RR of cancer in SAMs in Leeds was 31.56 (CI 18.6, 53.5; *P* < 0.01). The results of a systematic review [2], summarizing data for five population cohort studies and 4538 patients in total, demonstrated a RR of 17.29 (95% CI 11.08, 26.99). The difference in results may be due to the fact that these studies used more thorough reviews of cancer cases and diagnoses, whereas single reports in the patient notes were considered sufficient in the present study. Furthermore, the present study did not account for confounding variables such as smoking and alcohol consumption as potential risk factors, because of the lack of relevant data in the included articles. Nevertheless, our data support the existing literature that there is a significant increase in the risk of cancer associated with SAMs [2, 11, 14, 25]. The average time frame of 3 years between a diagnosis of SAMs and a cancer diagnosis also corresponded with previous data [2]; however, the RRs were calculated by including cancers reported within 10 years before a diagnosis of SAMs. Comparing DM and PM, the proportion of cancer was 60% (6/10 cases) and 8.3% (1/12 cases), respectively; an RR of 7.2 (CI 1.03, 50.28). These results are consistent with a meta-analysis systematic review [25], recent individual studies [11, 14–16] and a recent systematic review [2]. However, this study found an RR of 86 (CI 51.62, 143.56) comparing DM individually with the denominator population. This is considerably higher than in previous studies, which is likely due to 32% of unspecified SAM cases in this study. It is possible that many of the unspecified cases were true DM or PM diagnoses. Therefore the RRs of individual subtypes are potentially overrepresentative and must be interpreted with some limitations. Despite this, the present study is consistent with previous research indicating that DM is found to be more strongly associated with cancer than PM. Due to few case numbers, it was deemed inappropriate to analyse the incidence or RR of separate cancer types in relation to SAMs.

Qiang *et al.* [2] hypothesized that the increased incidence of cancer in SAMs may be partially attributable to more comprehensive cancer screening in this population, particularly within the first year after diagnosis. However, previous research, and now the present study, has demonstrated an increased incidence of cancer in patients even prior to a diagnosis of SAMs, which suggests that there may be a true association between SAMs and cancer [29]. One potential explanation is that SAMs-specific autoantigens may be involved in neoplastic processes. These antigens are expressed at high levels in cancers that are associated with DM and PM [30]. To date, the pathogenesis of this association is still unknown and the hypothesis needs testing in a large cohort.

This study has some limitations. First, this was a single-centre, retrospective study with a small population size. This is partly due to the rarity of the studied disease. Nonetheless, compared with other referenced studies examining nationwide incidences, the present study is very limited when considering the incidence of individual subtypes. However, the city of Leeds was chosen as a focus, rather than the whole of Yorkshire, to accurately determine cases and provide more robust data acquisition. Using diagnostic criteria with a retrospective review may underestimate the true incidence due to missing data, as most criteria require several investigations, of which some information may be missing from patient records. As such, despite manually reviewing each case and requiring biopsy verification, 32% of SAMs diagnoses were unspecified by subtype. This was likely due to the histopathological focus of the method and incomplete data available on SAM-specific antibodies. It is evident that, at present, the process of defining histological features as part of the diagnostic criteria for SAMs is still debated and may produce an underestimation of results due to the heterogeneity of disease presentation and variable yield of muscle biopsy [24, 31–33]. On the other hand, a strength in manually reviewing records and requiring biopsy verification of cases is a higher specificity of results compared with studies using coding searches alone.

## Conclusion

In summary, the mean incidence of adult SAMs in Leeds was 7.42 (CI 4.81, 10.03)/1 000 000 py; it appears to be more common in older females and is in keeping with estimates from other international studies. With a better understanding of the disease and validation of a single, universally accepted diagnostic criteria, it is hoped that future studies will clarify the uncertainties of inflammatory myopathies. This study provides data to complement existing reviews and supports the practice of cancer screening and long-term surveillance, with an emphasis on the need to screen for malignancy over time in patients with SAMs, in particular DM. The pathogenesis of this relationship is still unknown and requires investigating, with further insights into differences between subtypes. The present study underscores the difficult challenges in the epidemiological study of SAMs but may nevertheless provide useful clues for the comprehension of SAMs.

*Funding*: No specific funding was received from any bodies in the public, commercial or not-for-profit sectors to carry out the work described in this article.

*Disclosure statement*: The authors have declared no conflicts of interest.

## Data availability statement

The datasets generated during and/or analysed during the current study are available from the corresponding author upon reasonable request.

## Supplementary data

Supplementary data are available at *Rheumatology Advances in Practice* online.

## Supplementary Material

rkac023_Supplementary_Data

## References

[1] Dalakas MC. Inflammatory muscle diseases. N Engl J Med 2015;373:393–4. 10.1056/NEJMc1506827 26200989

[2] Qiang JK , Kim WB, Baibergenova A, Alhusayen R. Risk of malignancy in dermatomyositis and polymyositis: a systematic review and meta-analysis. J Cutan Med Surg 2017;21:131–6. 27534779 10.1177/1203475416665601

[3] Barsotti S , Lundberg IE. Myositis an evolving spectrum of disease. Immunol Med 2018;41:46–54. 30938263 10.1080/13497413.2018.1481571

[4] Pinto B , Janardana R, Nadig R et al Comparison of the 2017 EULAR/ACR criteria with Bohan and Peter criteria for the classification of idiopathic inflammatory myopathies. Clin Rheumatol 2019;38:1931–4. 30903308 10.1007/s10067-019-04512-6

[5] Lundberg IE , Tjarnlund A, Bottai M et al 2017 European League Against Rheumatism/American College of Rheumatology classification criteria for adult and juvenile idiopathic inflammatory myopathies and their major subgroups. Arthritis Rheumatol 2017;69:2271–82. 29106061 10.1002/art.40320 PMC5846474

[6] Svensson J , Arkema EV, Lundberg IE, Holmqvist M. Incidence and prevalence of idiopathic inflammatory myopathies in Sweden: a nationwide population-based study. Rheumatology (Oxford) 2017;56:802–10. 28160487 10.1093/rheumatology/kew503

[7] MedlinePlus. Idiopathic inflammatory myopathy. https://medlineplus.gov/genetics/condition/idiopathic-inflammatory-myopathy/ (6 November 2020, date last accessed).

[8] Parker MJS , Oldroyd A, Roberts ME et al Increasing incidence of adult idiopathic inflammatory myopathies in the city of Salford, UK: a 10-year epidemiological study. Rheumatol Adv Pract 2018;2:rky035. 31431976 10.1093/rap/rky035 PMC6649983

[9] Azuma K , Yamada H, Ohkubo M et al Incidence and predictive factors for malignancies in 136 Japanese patients with dermatomyositis, polymyositis and clinically amyopathic dermatomyositis. Mod Rheumatol 2011;21:178–83. 20922453 10.1007/s10165-010-0362-y

[10] Chen YJ , Wu CY, Huang YL et al Cancer risks of dermatomyositis and polymyositis: a nationwide cohort study in Taiwan. Arthritis Res Ther 2010;12:R70. 20398365 10.1186/ar2987 PMC2888225

[11] Hill CL , Zhang Y, Sigurgeirsson B et al Frequency of specific cancer types in dermatomyositis and polymyositis: a population-based study. Lancet 2001;357:96–100. 11197446 10.1016/S0140-6736(00)03540-6

[12] Bowerman K , Pearson DR, Okawa J, Werth VP. Malignancy in dermatomyositis: a retrospective study of 201 patients seen at the University of Pennsylvania. J Am Acad Dermatol 2020;83:117–22. 32135206 10.1016/j.jaad.2020.02.061

[13] Dobloug GC , Garen T, Brunborg C, Gran JT, Molberg O. Survival and cancer risk in an unselected and complete Norwegian idiopathic inflammatory myopathy cohort. Semin Arthritis Rheum 2015;45:301–8. 26190563 10.1016/j.semarthrit.2015.06.005

[14] Huang YL , Chen YJ, Lin MW et al Malignancies associated with dermatomyositis and polymyositis in Taiwan: a nationwide population-based study. Br J Dermatol 2009;161:854–60. 19558555 10.1111/j.1365-2133.2009.09274.x

[15] Limaye V , Luke C, Tucker G et al The incidence and associations of malignancy in a large cohort of patients with biopsy-determined idiopathic inflammatory myositis. Rheumatol Int 2013;33:965–71. 22833242 10.1007/s00296-012-2489-y

[16] Stockton D , Doherty VR, Brewster DH. Risk of cancer in patients with dermatomyositis or polymyositis, and follow-up implications: a Scottish population-based cohort study. Br J Cancer 2001;85:41–5. 11437400 10.1054/bjoc.2001.1699 PMC2363903

[17] Liu Y , Xu L, Wu H et al Characteristics and predictors of malignancy in dermatomyositis: analysis of 239 patients from northern China. Oncol Lett 2018;16:5960–8. 30344746 10.3892/ol.2018.9409 PMC6176340

[18] Amoura Z , Duhaut P, Huong DLT et al Tumor antigen markers for the detection of solid cancers in inflammatory myopathies. Cancer Epidemiol Biomarkers Prev 2005;14:1279–82. 15894686 10.1158/1055-9965.EPI-04-0624

[19] Buchbinder R , Forbes A, Hall S, Dennett X, Giles G. Incidence of malignant disease in biopsy-proven inflammatory myopathy: a population-based cohort study. Ann Intern Med 2001;134:1087–95. 11412048 10.7326/0003-4819-134-12-200106190-00008

[20] Meyer A , Meyer N, Schaeffer M et al Incidence and prevalence of inflammatory myopathies: a systematic review. Rheumatology (Oxford) 2015;54:50–63. 25065005 10.1093/rheumatology/keu289

[21] Patrick M , Buchbinder R, Jolley D, Dennett X, Buchanan R. Incidence of inflammatory myopathies in Victoria, Australia, and evidence of spatial clustering. J Rheumatol 1999;26:1094–100. 10332974

[22] Weitoft T. Occurrence of polymyositis in the county of Gavleborg, Sweden. Scand J Rheumatol 1997;26:104–6. 9137324 10.3109/03009749709115827

[23] Dobloug C , Garen T, Bitter H et al Prevalence and clinical characteristics of adult polymyositis and dermatomyositis; data from a large and unselected Norwegian cohort. Ann Rheum Dis 2015;74:1551–6. 24695011 10.1136/annrheumdis-2013-205127

[24] Tan JA , Roberts-Thomson PJ, Blumbergs P et al Incidence and prevalence of idiopathic inflammatory myopathies in South Australia: a 30-year epidemiologic study of histology-proven cases. Int J Rheum Dis 2013;16:331–8. 23981756 10.1111/j.1756-185X.2011.01669.x

[25] Oldroyd AGS , Allard AB, Callen JP et al A systematic review and meta-analysis to inform cancer screening guidelines in idiopathic inflammatory myopathies. Rheumatology (Oxford) 2021;60:2615–28. 33599244 10.1093/rheumatology/keab166 PMC8213426

[26] Lundberg IE , Miller FW, Tjarnlund A, Bottai M. Diagnosis and classification of idiopathic inflammatory myopathies. J Intern Med 2016;280:39–51. 27320359 10.1111/joim.12524 PMC5021058

[27] Troyanov Y , Targoff IN, Tremblay JL et al Novel classification of idiopathic inflammatory myopathies based on overlap syndrome features and autoantibodies: analysis of 100 French Canadian patients. Medicine (Baltimore) 2005;84:231–49. 16010208 10.1097/01.md.0000173991.74008.b0

[28] Chung SW , Yoo IS, Kim J et al Comparison of the 2017 EULAR/ACR criteria with clinicoserologic criteria for the classification of idiopathic inflammatory myopathies in Korean patients. Yonsei Med J 2021;62:424–30. 33908213 10.3349/ymj.2021.62.5.424 PMC8084694

[29] Liu WC , Ho M, Koh WP et al An 11-year review of dermatomyositis in Asian patients. Ann Acad Med Singapore 2010;39:843–7. 21165524

[30] Casciola-Rosen L , Nagaraju K, Plotz P et al Enhanced autoantigen expression in regenerating muscle cells in idiopathic inflammatory myopathy. J Exp Med 2005;201:591–601. 15728237 10.1084/jem.20041367 PMC2213068

[31] Mammen AL. Dermatomyositis and polymyositis: clinical presentation, autoantibodies, and pathogenesis. Ann N Y Acad Sci 2010;1184:134–53. 20146695 10.1111/j.1749-6632.2009.05119.x

[32] Mastaglia FL , Phillips BA. Idiopathic inflammatory myopathies: epidemiology, classification, and diagnostic criteria. Rheum Dis Clin North Am 2002;28:723–41. 12510664 10.1016/s0889-857x(02)00021-2

[33] Targoff IN , Miller FW, Medsger TA Jr, Oddis CV. Classification criteria for the idiopathic inflammatory myopathies. Curr Opin Rheumatol 1997;9:527–35. 9375282 10.1097/00002281-199711000-00008

