# Supplementary material for: Incidence of systemic autoimmune myopathies and their risk of cancer in Leeds, UK: an 11-year epidemiological study
Source: Rheumatol Adv Pract. 2022 Mar 28;6(1):rkac023. doi: 10.1093/rap/rkac023 (PMC9024320; doi:10.1093/rap/rkac023)
Supplement: rkac023_Supplementary_Data [file rkac023_supplementary_data.docx]

**Supplementary Data – Jauniaux/Ismail**

**Supplementary Table S1:** The annual population of the Leeds Care Group, UK

| Year | Males | Females |
| --- | --- | --- |
| 2020 | 306749 | 323996 |
| 2019 | 302256 | 321461 |
| 2018 | 300885 | 320133 |
| 2017 | 299995 | 318583 |
| 2016 | 299348 | 316920 |
| 2015 | 296299 | 314326 |
| 2014 | 293504 | 311450 |
| 2013 | 292341 | 309961 |
| 2012 | 291449 | 309269 |
| 2011 | 288869 | 306931 |
| 2010 | 288692 | 304677 |

**Supplementary Table S2:** Individual case characteristics

| Sex | Clinical Diagnosis | Antibody findings | Any malignancies found within 10y before diagnosis | Any malignancies found within 10y after diagnosis | Comorbidities |
| --- | --- | --- | --- | --- | --- |
| F | Not specified |  |  |  | cutaneous systemic sclerosis |
| F | IBM |  |  |  | IHD, AF, HTN |
| F | PM | Anti Ro (52), Anti Jo-1 |  |  | ILD |
| F | DM |  |  |  | n/a |
| F | Not specified (**2011**) | Anti Ro (52), ANCA |  | Pancreas (**2015**),  Lymphatic (**2013**) | vasculitis, trigeminal neuralgia, ILD, RA, raynaud, endometriosis, scleroderma, ostepaenia, HTN |
| F | Necrotising | Anti Ro (52) |  |  | neuropathic bladder, sjogren's, coeliac |
| F | DM (**2012**) |  |  | Colon (**2016**),  SCC skin (**2020**) | scleroderma, coronary artery disease, osteoporosis |
| F | PM | Rheumatoid Factor |  |  | essential HTN, pulmonary HTN, ostopaenia |
| F | Not specified |  |  |  | n/a |
| F | IBM |  |  |  | n/a |
| F | Not specified |  |  |  | uveitis |
| M | PM |  |  |  | aortic stenosis, complete heart block, HTN |
| F | DM (**2013**) | Anti La, Anti Sm/RNP, ANCA | BCC skin  (**2010)** | Breast (**2014**) | T2DM, diverticular disease, essential HTN, hypothyroidism |
| M | DM (**2013**) | Anti Ro (52), Anti Jo-1 |  | Colon (**2018**) | calcinosis, pulmonary fibrosis, thrombocytopaenia, normocytic anaemia |
| M | Necrotising |  |  |  | hepatitis B, gout |
| M | IBM |  |  |  | n/a |
| F | PM (**2013**) |  |  | Pancreas (**2016**) | systemic sclerosis, osteoarthritis |
| F | IBM |  |  |  | oesophagitis, osteoporosis |
| M | PM |  |  |  | thrombocytopaenia, normocytic anaemia, IHD, HTN, T2DM |
| F | Not specified |  |  |  | sarcoidosis, gout |
| M | PM | Anti Ro (52), Anti Jo-1 |  |  | ILD (anti-synthetase syndrome), osteoarthritis |
| M | PM | ANCA |  |  | ILD, parkinson’s, HTN |
| F | DM (**2015**) |  | Endometrium  (**2012**) |  | scleroderma |
| F | DM | Anti Sm/RNP, Anti RNP 68, Anti Centromere |  |  | n/a |
| F | PM |  |  |  | ILD |
| F | DM |  | Colon (**n/a**) |  | parkinson's |
| F | DM |  |  |  | n/a |
| M | IBM | Anti Ro (52) |  |  | HTN, gout |
| F | Not specified (**2018**) | DNA Ab | BCC skin (**2013**) |  | PVD |
| M | Not specified (**2018**) |  | Colon (**2016**) |  | anaemia, T2DM, eczema |
| F | IBM |  |  |  | n/a |
| M | DM (**2018**) | DNA Ab, ANCA, Anti CCP |  | Liver (**2018**) | raynaud's, RA, hepatitis, ILD, NAFLD |
| M | PM | Anti Scl-70 |  |  | Huntington's |
| M | ***Statin-induced*** |  |  |  | hypothyroidism, HTN, COPD, vitiligo, T2DM |
| M | Not specified |  |  |  | n/a |
| F | PM | Anti Ro (52) |  |  | hypothyroidism, asthma |
| F | ***Statin-induced*** | DNA Ab |  |  | osteoarthritis, COPD |
| F | Not specified |  |  |  | epilepsy |
| M | ***Statin-induced*** |  |  |  | rhabdomyolysis needing dialysis |
| M | PM |  |  |  | n/a |
| M | Not specified | Anti Ro (60), Anti Ro (52), Anti Chromatin, ANCA |  |  | MOF |
| M | ***Statin-induced*** |  |  |  | HTN, amyloidosis, neuropathy |
| M | Not specified |  |  |  | emphysema, IHD |
| M | PM |  |  |  | HTN, Asthma |
| M | Not specified |  |  |  | n/a |
| F | DM | ANCA |  |  | T1DM |
| M | Not specified |  |  |  | n/a |
| M | Not specified (**2020**) |  |  | Prostate (**2020**) | polymyalgia, essential hypertension |
| F | Not specified |  |  |  | hepatic steatosis, osteopaenia, chronic pancreatitis |
| M | Not specified | Anti Ro (52) |  |  | mitral valve regurgitation, IHD, HTN, gout, IHD, cervical spinal stenosis |

IBM: Inclusion body myositis, PM: Polymyositis, DM: Dermatomyositis, BCC: Basal cell carcinoma, SCC: Squamous cell carcinoma, IHD: Ischaemic heart disease, AF: Atrial fibrillation, HTN: Hypertension, RA: Rheumatoid arthritis, T2DM: Type 2 diabetes mellitus, ILD: Interstitial lung disease, PVD: Peripheral vascular disease, NAFLD: Non-alcoholic fatty liver disease, COPD: Chronic obstructive pulmonary disease, MOF: multi-organ failure.

**Supplementary Table S3:** Comorbidities found in SAM and SAM sub-types

| Comorbidities | DM | PM | IBM | Other | SAMs overall |
| --- | --- | --- | --- | --- | --- |
| Asthma | 0 | **2** | 0 | 0 | **2** |
| HTN | 0 | **5** | **2** | **5** | **12** |
| ILD | 0 | **4** | 0 | **1** | **5** |
| Scleroderma | **2** | 0 | 0 | **1** | **3** |
| Parkinson's | **1** | **1** | 0 | 0 | **2** |
| OA/RA | 0 | **2** | 0 | **2** | **4** |
| Systemic sclerosis | 0 | **1** | 0 | **1** | **2** |
| Gout | 0 | 0 | **1** | **3** | **4** |
| Individual Malignancies* | **8** | **1** | 0 | **5** | **14** |
| Cases with at least one malignancy | **6** | **1** | 0 | **4** | **11** |
| Details | BCC skin, SCC skin, colon (n =3), breast, liver, endometrium | Pancreas |  | Pancreas, lymphatic, BCC skin, colon, prostate |  |

*3 cases had two reported malignancies

DM: Dermatomyositis, PM: Polymyositis, IBM: Inclusion body myositis, SAM: systemic autoimmune myopathies, HTN: Hypertension, ILD: Interstitial lung disease, OA: Osteoarthritis, RA: Rheumatoid arthritis, BCC: Basal cell carcinoma, SCC: Squamous cell carcinoma

**Supplementary Table S4:** Annual SAM cases and incidence rates per 1 million population

| Year | PM | DM | IBM | Other | SAM | Inc. rate PM | Inc. rate DM | Inc. rate IBM | Inc. rate Other | Inc. rate SAM |
| --- | --- | --- | --- | --- | --- | --- | --- | --- | --- | --- |
| 2020 | **0** | **1** | **0** | **5** | **6** | 0.00 | 1.59 | 0.00 | 7.93 | **9.51** |
| 2019 | **3** | **0** | **0** | **6** | **9** | 4.81 | 0.00 | 0.00 | 9.62 | **14.43** |
| 2018 | **1** | **1** | **1** | **4** | **7** | 1.61 | 1.61 | 1.61 | 6.44 | **11.27** |
| 2017 | **0** | **1** | **1** | **0** | **2** | 0.00 | 1.62 | 1.62 | 0.00 | **3.23** |
| 2016 | **0** | **1** | **0** | **0** | **1** | 0.00 | 1.62 | 0.00 | 0.00 | **1.62** |
| 2015 | **1** | **2** | **0** | **0** | **3** | 1.64 | 3.28 | 0.00 | 0.00 | **4.91** |
| 2014 | **4** | **0** | **2** | **1** | **7** | 6.61 | 0.00 | 3.31 | 1.65 | **11.57** |
| 2013 | **1** | **2** | **1** | **3** | **7** | 1.66 | 3.32 | 1.66 | 4.98 | **11.62** |
| 2012 | **1** | **1** | **0** | **0** | **2** | 1.66 | 1.66 | 0.00 | 0.00 | **3.33** |
| 2011 | **0** | **0** | **0** | **2** | **2** | 0.00 | 0.00 | 0.00 | 3.36 | **3.36** |
| 2010 | **1** | **1** | **1** | **1** | **4** | 1.69 | 1.69 | 1.69 | 1.69 | **6.74** |
| Average | **1.1** | **0.9** | **0.6** | **2** | **4.6** | 1.79 (0.5-3) | 1.49 (0.81-2.17) | 0.9 (0.23-1.57) | 3.24 (1.17-5.31) | **7.42 (4.81-10.03)** |

Incidence rate (Inc. rate) measured per 1 million population.

PM: Polymyositis, DM: Dermatomyositis, IBM: Inclusion body myositis, SAM: systemic autoimmune myopathies
